# Body Temperature Measurements for Metabolic Phenotyping in Mice

**DOI:** 10.3389/fphys.2017.00520

**Published:** 2017-07-31

**Authors:** Carola W. Meyer, Youichirou Ootsuka, Andrej A. Romanovsky

**Affiliations:** ^1^Department of Pharmacology, Max-Planck Institute for Heart and Lung Research Bad Nauheim, Germany; ^2^Centre for Neuroscience, School of Medicine, Flinders University of South Australia Adelaide, SA, Australia; ^3^FeverLab, St. Joseph's Hospital and Medical Center Phoenix, AZ, United States

**Keywords:** mouse, phenotyping, body temperature, thermography, metabolism, telemetric recordings, mouse models

## Abstract

Endothermic organisms rely on tightly balanced energy budgets to maintain a regulated body temperature and body mass. Metabolic phenotyping of mice, therefore, often includes the recording of body temperature. Thermometry in mice is conducted at various sites, using various devices and measurement practices, ranging from single-time probing to continuous temperature imaging. Whilst there is broad agreement that body temperature data is of value, procedural considerations of body temperature measurements in the context of metabolic phenotyping are missing. Here, we provide an overview of the various methods currently available for gathering body temperature data from mice. We explore the scope and limitations of thermometry in mice, with the hope of assisting researchers in the selection of appropriate approaches, and conditions, for comprehensive mouse phenotypic analyses.

## Key points

- Rectal probing is subject to procedural bias. This method is suitable for first-line phenotyping, provided probe depth and measurement duration are standardized. It is also useful for detecting individuals with out-of-range body temperatures (during hypothermia, torpor).
- The colonic temperature attained by inserting the probe >2 cm deep is a measure of deep (core) body temperature.
- IR imaging of the skin is useful for detecting heat leaks and autonomous thermoregulatory alterations, but it does not measure body temperature.
- Temperature of the hairy or shaved skin covering the inter-scapular brown adipose tissue can be used as a measure of BAT thermogenesis. However, obtaining such measurements of sufficient quality is very difficult, and interpreting them can be tricky. Temperature differences between the inter-scapular and lumbar areas can be a better measure of the thermogenic activity of inter-scapular brown adipose tissue.
- Implanted probes for precise determination of BAT temperature (changes) should be fixed close to the Sulzer's vein. For measurement of BAT thermogenesis, core body temperature and BAT temperature should be recorded simultaneously.
- Tail temperature is suitable to compare the presence or absence of vasoconstriction or vasodilation.
- Continuous, longitudinal monitoring of core body temperature is preferred over single probing, as the readings are taken in a non-invasive, physiological context.
- Combining core body temperature measurements with metabolic rate measurements yields insights into the interplay between heat production and heat loss (thermal conductance), potentially revealing novel thermoregulatory phenotypes.

## Introduction

Precise phenotyping of mice strains and genetically modified mice has become increasingly important for revealing correlations and inferring causality amongst specific physiological pathways. Important targets in mouse phenotyping include changes in energy balance, which can result in altered body composition (Tschöp et al., 2012; Rozman et al., 2014). The majority of studies targeting energy balance involve measurements of body mass, fat mass, and food (energy) intake (Moir et al., 2016). Many authors also report energy expenditure by using indirect calorimetry (Speakman, 2013; Meyer et al., 2015). In addition, body temperature is often measured.

At present, mouse body temperature data for metabolic phenotyping is mainly obtained by one of three methods: (1) inserting a probe into the rectum (or, less typically, into the sigmoid colon); (2) measuring temperature in the abdominal cavity or in the subcutaneous compartment with a pre-implanted probe; or (3) measuring surface temperature with infrared (IR) thermography (e.g., from the tail, trunk areas, external auditory meatus, or eyes). In many cases, thermometry data obtained by any of these methods is presented as a single (often the mean) value labeled “body temperature.” Unfortunately, this simplistic approach ignores the fact that the body of a mouse is thermally heterogeneous - and that all sites produce different output values. For example, IR thermography of a mouse tail provides information about the vasomotor tone of the tail vasculature, whereas colonic thermometry produces a value of deep body temperature. This creates a need for the appropriate labeling of temperature data. Condensing complex data into a single mean value also dismisses the pronounced circadian rhythms of body temperature in mice and, in general, ignores the fact that body temperature is constantly affected by changes in both the external and internal environments (Gordon, 1993).

We think that the method of thermometry (i.e., where and how body temperature is measured) and the experimental conditions (e.g., ambient temperature, whether or not the animals are restrained, and whether or not they are acclimated to the experimental setups and procedures) are of enormous importance for the interpretation of any body temperature data obtained during metabolic phenotyping of genetically modified mice. Below, we overview the main methods used for thermometry at different sites of the body in the mouse (with occasional reference to the rat) and address the utility of these methods in the context of mouse metabolic phenotyping.

### Rectal (or colonic) thermometry

Rectal thermometry is a common method of measuring body temperature in rodents. It involves inserting a small-diameter temperature probe through the anus. The temperature-sensitive element of the probe is either a thermistor, a resistance temperature detector (RTD), or the “hot” (active) junction of a thermocouple (Box 1). As the core of the body is warmer than the shell, the body temperature value obtained with a rectal probe critically depends on the insertion depth. In adult mice, an insertion depth of >2 cm will yield colonic temperatures. In fact, the temperature measured by this method is one of highest temperatures in the body of endotherms, corresponding to deep (core) body temperature (Donhoffer, 1980).

Box 1 **Thermocouples, thermistors, and RTDs.**

A thermocouple is formed by two dissimilar metals. The voltage produced by such a junction is temperature-dependent. Different types of thermocouples use different metal combinations and, therefore, have different characteristics. By convention, the color of the thermocouple connector identifies the type. For most physiological purposes, including rectal thermometry, copper-constantan (T-type) thermocouples are used; their plugs are usually made of blue plastic. The response time of these temperature probes is often around 0.5 s, but it can vary widely based on the probe size and insulating materials used. The accuracy of thermocouple probes can be rather high (<0.1°C), but in most commercially available devices for rectal thermometry, it is somewhere between 0.1 and 0.5°C.

Thermistors and the so-called RTDs (resistance temperature detectors) are used to measure temperature because their electrical resistance depends on temperature. Thermistors are generally made from certain metal oxides, often incased in ceramic, and their resistance decreases with increasing temperatures. RTDs are made of metals, such as platinum and nickel, and their resistance increases with temperature. In general, RTDs have technical characteristics that are superior to those of thermistors and thermocouples, but these minor technical differences are of little relevance to the typical tasks of rectal thermometry in mice. RTDs are also more expensive.

The material of the probe shaft is relevant when environmental parameters are significantly deviating from physiological, i.e., during cold exposure. If the metal holder of the probe is short, it is able to transfer energy to the sensor, hence pushing the readings toward cooler values in cold. In this case, non-metal shafts are preferred in order to reduce bias.

Less-deep insertion of the probe results in somewhat lower and more variable readings, corresponding to rectal temperatures. Despite the fact that rectal temperature is somewhat inferior to colonic temperature; it is still a valid measure. Rectal thermometry is the simplest, and sometimes lowest-cost, method for obtaining body temperature data in conscious mice. It is also the lowest-impact method, as the procedure is fast and painless for mice, and no surgery is required. To obtain a rectal temperature, the mouse is usually hand-restrained and placed on a horizontal surface, e.g., a cage lid. The tail is then lifted, and a probe (covered with Vaseline) is gently inserted into the rectum to a fixed depth (typically, up to 2 cm). Although different laboratories and manufacturers recommend different insertion depths, it is critical that the depth be exactly the same for each measurement to reduce within-group variability. For comparative purposes, insertion depth should be routinely reported in publications involving rectal thermometry.

The time of rectal readings depends on the time constant of the probe and is often specified by the manufacturer. If the reading is taken too fast, the value obtained underestimates the real temperature. However, waiting too long may also create a problem as body temperature increases rapidly (i.e., within seconds) as part of the stress response of the mouse to being handled and fixated (Clement et al., 1989). Hence, rectal body temperature readings obtained at longer time periods are more likely to be “contaminated” by stress hyperthermia. Acute stress-induced increases in body temperature can be greatly alleviated if the mice are trained to the measurement procedure and to restraint (Garami et al., 2011). Rodents are readily adaptable to confinement, and-when habituated-incur neither a stress fever (Romanovsky et al., 1998) nor show any other signs of stress (Hashimoto et al., 1988; Melia et al., 1994; Stamp and Herbert, 1999).

Rectal probing is particularly useful in diagnosing body temperatures outside the normal range, e.g., during conditions of torpor and hypothermia (Haemmerle et al., 2006). Provided that variations in probe depth, mouse age and sex, as well as other factors impacting body temperature are controlled for, rectal thermometry can also be used for first-line phenotyping of cohorts of genetically modified mice (Willershäuser et al., 2012). Because the method is prone to environmental and procedural variations (Zethof et al., 1994), it is more useful for screening for large effects on body temperature rather than for studying mechanisms of effects on body temperature - especially if the effects are moderate or small. Assuming an insertion depth of 2 cm and a standard deviation of 0.4°C for inbred C57BL/6J mice (Willershäuser et al., 2012), detecting a moderate difference of 0.5°C in rectal temperature at *p* < 0.05 and a power of 80% (two-sided *t*-test) would require the use of at least 12 animals per group.

### Wireless measurements of body temperature using implanted probes

Wireless monitoring of temperature can be achieved using probes that are firmly anchored to the inside of the body to obtain temperature data from freely-moving, conscious animals. Table 1 gives an overview of the current products available for measuring internal temperatures in unrestrained mice and other small mammals. Some of the probes not only provide temperature readings, but also enable the measurement of gross motor activity. The devices listed are transmitters, transponders and data loggers (Boxes 2–4). In addition, novel implantable transponder-logger hybrids have been made available to enable the identification and monitoring of multiple animals in one cage (see Table 1). This relieves the constraint to house animals individually for measuring body temperature, thus allowing for no-contact thermometry in a social context.

**Table 1:** Temperature probes for measuring body temperatures in unrestrained mice.

**Product name**	**Type**	**Mass (g)**	**Volume (cm^3^)**	**Simultaneous acquisition in multiple animals**	**Average battery life^d^ (months)**	**Manufacturer URL**
DSI PhysioTel TA-F10^c^	Transmitter	1.6	1.1	No	6	www.datasci.com
Anipill	Transmitter	1.7	1.2	Yes	7	www.datasci.com
E-Mitter Series 3000 XM-FH^c^	Transponder	1.6	1.1	No	Battery-free	http://www.minimitter.com/www.respironics.com^a^
G2 E- Mitter^c^	Transponder	1.1	0.6	No	Battery-free	www.starrlifesciences.com^b^
IPTT-300	Transponder	0.1	0.1	No	Battery-free	www.bmds.com
SubCue Mini	Logger	2.5	1.5	Yes	30	www.subcue.com
DST nano-T	Logger	1.0	0.5	Yes	14	www.star-oddi.com
DST nano RF-T	Transponder-logger	1.3	0.6	Yes	12	www.star-oddi.com
XS Stellar telemetry^c^	Transponder-logger	2.5	1.5	Yes	5	www.tse-systems.com
Mouse Monitor™ C19BTA^c^	Transponder-logger	2.7	1.9	Yes	0.5 rechargeable	www.indusinstruments.com

a *This product is no longer commercially available, but is still used in some laboratories*.

b *In 2013, STARR Life Sciences (international distributor: Harvard Apparatus Ltd) acquired the VitalView/E-Mitter product line from Philips Respironics*.

c *This product also collects information on gross motor activity*.

d *Battery life depends on sampling frequency. Battery-free devices require no refurbishment. Their maximal lifetime is variable (usually >2 years)*.

Box 2 **Transponders.**

A transponder is an electronic device that can receive radiofrequency signals in response to predetermined signals. Hence, it can function as both a transmitter and a responder. If used for animal identification purpose, its function is to send out an identifier signal when an outside signal requests identification. A transponder consists of a coil antenna coupled to an integrated circuit chip, both covered by biocompatible glass or plastic. For temperature-measuring transponders, the chip includes a thermistor to measure temperature and a memory unit to store temperature data. An example of a transponder, IPTT-300, is listed in Table 1. This is a low-accuracy (0.5°C) device used mostly in large-scale animal husbandry operations, where some rough estimate of body temperature is obtained “for free” along with the animal's identifier. A main advantage of transponders is the ease of their implantation (using a syringe-like injector) and their battery-free operation. Once implanted, a transponder can provide temperature data for the entire life of the animal.

Box 3 **Telemetry transmitters.**

A transmitter is an electronic device that generates and amplifies a carrier wave, modulates it according to its temperature, and broadcasts the resulting signal from an antenna. Signal modulation by temperature is typically achieved through changes in either the frequency (frequency modulated, FM) or the amplitude (amplitude modulated, AM). For implantable transmitters used to measure body temperature, all the electronics involved in sensing temperature, modulating and transmitting the signal are hidden inside a miniature biocompatible capsule. Several examples of transmitters are listed in Table 1. Their accuracy is generally greater than 0.2°C. For those experimental conditions where a radio signal from a transmitter implanted in an animal can be easily detected by a receiver, transmitter-based telemetry is often a method of choice for real-time thermometry.

Box 4 **Data loggers.**

A temperature data logger is an electronic device that stores temperature signals in digital form on silicon or wax-coated memory chips integrated with a temperature probe. The device is programmed and implanted into an animal. The temperature information can be read only after the logger is removed from the animal, which usually happens at the end of the experiment when the animal is euthanized. The sampling frequency is set during programming, and the amount of data that can be stored and retrieved depends on the memory size. For example, if the memory size is 2,000 data points, the logger can store the data for ~1.4 days of recordings at 1-min sampling, or for about 85 days of recordings at 1-h sampling. The major advantage of data loggers is that they can be used under those conditions that do not permit communication between a transmitter and receiver, e.g., in a field experiment, or when an animal is placed in an environment impermeable to radio waves, such as in some designs of a thermogradient apparatus (Almeida et al., 2006; Garami et al., 2011).

Owing to their size, miniature transponders, e.g., IPTT-300, are typically implanted subcutaneously. They represent advanced versions of animal identification chips and, as such, provide subcutaneous temperature measurements as an “added bonus.” At most locations (e.g., on the back of the animal), the subcutaneous temperature determined by a transponder will be close to the temperatures of the adjacent hairy (non-glabrous) skin (Romanovsky, 2014). Subcutaneous temperatures can vary widely and are strongly affected by the ambient temperature. They cannot serve as a measure of deep body temperature.

**Figure 1:**
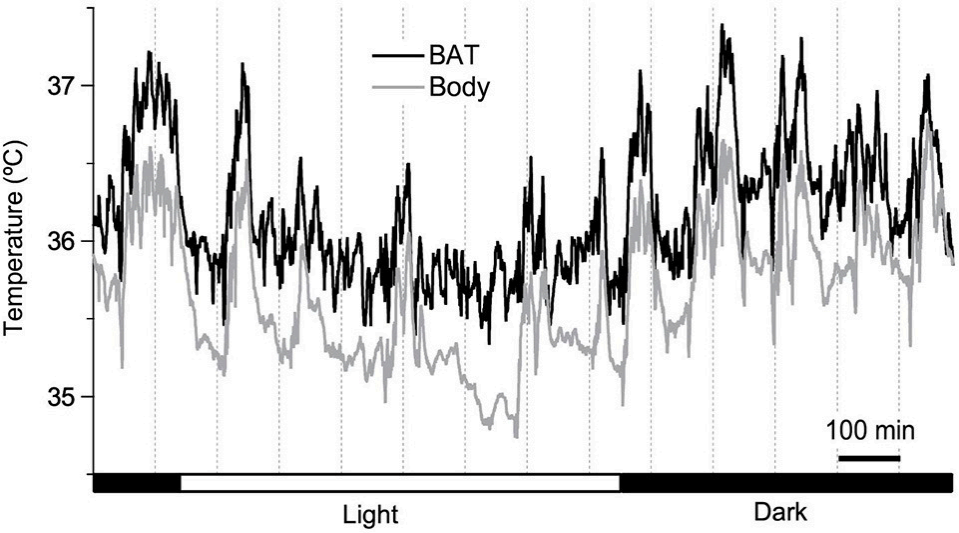
Twenty four-hour brown adipose tissue (BAT) thermometry and abdominal body temperature readings in a freely-moving C57BL/6 mouse maintained at 26°C. Abdominal temperature was measured using an implanted telemetry transmitter (DSI, ETA-F10). BAT temperature was measured using a thermistor (NTH5G10P, muRata, Kyoto, Japan) implanted between the BAT and the underlying muscle layer in the inter-scapular region near Sulzer's vein, and was connected to a swivel. The resolution of readings was set to 1 Hz. Y. Ootsuka, unpublished data.

In some mouse studies, miniature transponders are implanted in proximity to the inter-scapular brown adipose tissue (BAT) depots (e.g., Bal et al., 2012; Gerhart-Hines et al., 2013; Muller et al., 2013; Lateef et al., 2014). Although some of these studies claim successful use of such implants for assessing the (change in) temperature of BAT (as a measure of thermogenic activity), we are skeptical about such applications and do not recommend them - considering the rather large size of the transponder in relation to the BAT tissue. There are also some technical concerns: when thermogenesis in BAT is activated, the generated heat is collected throughout the brown fat pads by venous blood and leaves the tissue through Sulzer's vein (Smith and Horwitz, 1969). The amount of heat generated is so high that it is sufficient to rapidly warm the entire body of an animal by a few degrees Celsius. Hence, in active BAT, a proper measurement of brown fat temperature is substantially (sometimes by more than 1°C) higher than core body temperature, as was shown in rats with thermocouples acutely implanted in the inter-scapular depots of brown fat immediately before an experiment (Szekely et al., 1973; Szekely and Szelenyi, 1979), and in some experiments using chronic implantation techniques (Romanovsky et al., 1997; Ootsuka et al., 2007; Almeida et al., 2012). In chronic experiments, it is difficult to achieve the quality of measurements that would allow one to accurately quantify this phenomenon. To achieve a high-quality BAT temperature measurement, a probe must be fixed in the immediate proximity of Sulzer's vein with special techniques (e.g., Almeida et al., 2012; and Figure 1).

Even when a probe is properly positioned near Sulzer's vein, BAT temperature can be influenced by temperature changes in the muscles underneath the BAT depots, and in the skin above. To control for these influences, at least partially, it is important to measure core body temperature at the same time. The difference between BAT and core body temperature, sometimes referred to as “BAT themogenic index,” can be used to assess BAT heat production (Almeida et al., 2012). Comparing the slope of an increase in BAT temperature during the initial phase of BAT thermogenesis with the slope of core body temperature is a way to assess the validity of BAT temperature measurements (Mohammed et al., 2014).

Most often, the probes listed in Table 1 are implanted into the peritoneal cavity to measure what is commonly referred to as “abdominal temperature.” Abdominal temperature is a valid measure of deep (core) body temperature. The associated surgical intervention involves a small midline incision through the skin, followed by an opening of the peritoneal cavity through the *linea alba*. In order to prevent probe migration in the peritoneal cavity, some devices have small loops for suture-anchoring to the abdominal wall. Alternatively, the probe is sometimes left free-floating in the peritoneal cavity, which increases the intra- and inter-animal variation in recorded temperature, and is not recommended.

**Figure 2:**
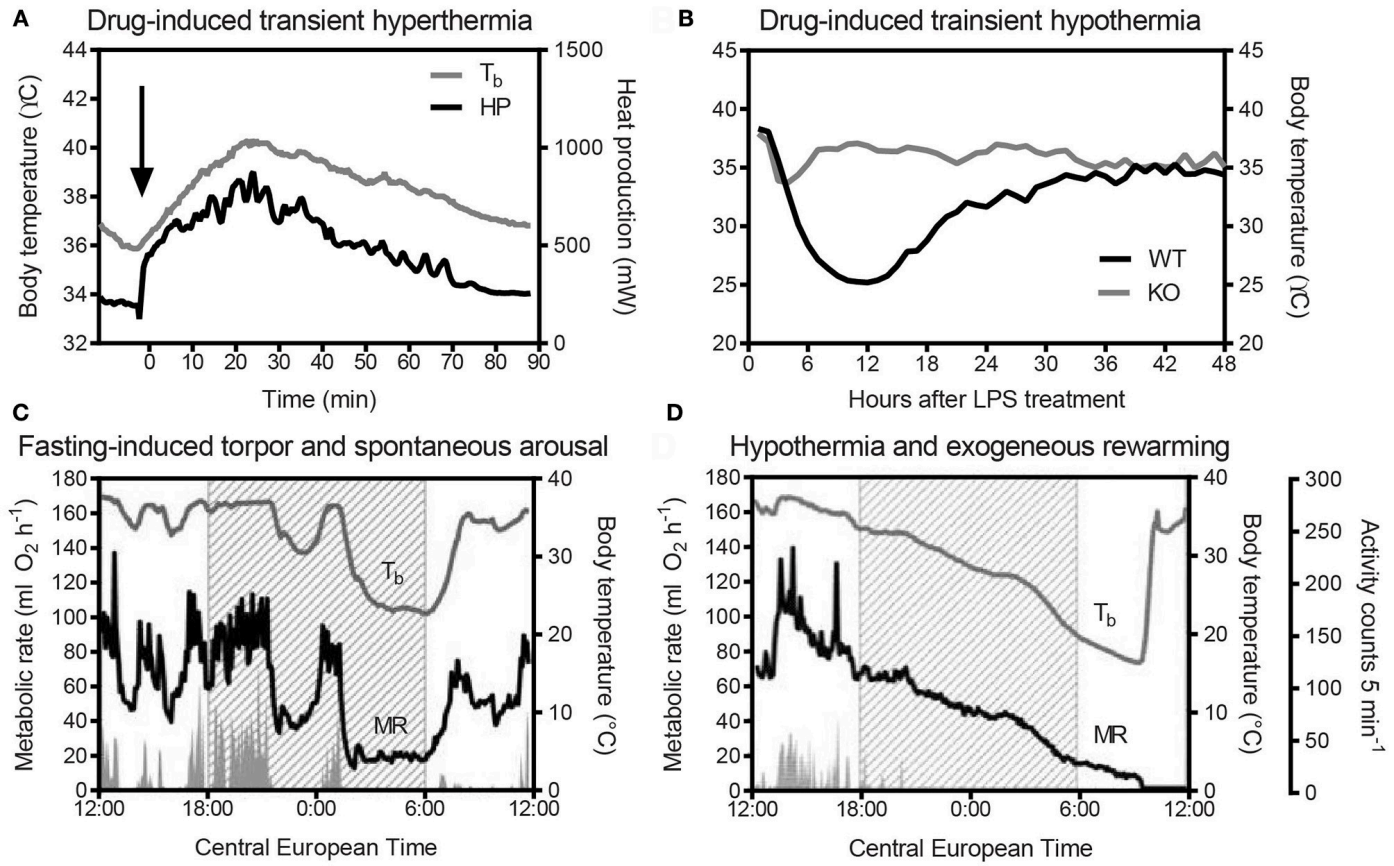
**(A)** Adrenergically-stimulated thermogenesis [heat production (HP)] following 1 mg/kg norepinephrine, s.c. (Arterenol, Merck; the arrow indicates the time-point of injection), and transient hyperthermia in a male AKR/J mouse kept at 30°C (N. Rink and C. W. Meyer; unpublished data). Body temperature (T_b_) of the individual was recorded with an intraperitoneally-implanted probe (MiniMitter, Sunriver, OR, USA) at a frequency of 2 min. HP was determined in parallel using indirect calorimetry (Meyer et al., 2015). **(B)** Genotype-dependent variation in lipopolysaccharide (LPS)-induced (15 μg/g, i.p.) transient hypothermia in mice, as determined by an intra-abdominal temperature sensor (DSI TA-F10). KO: knockout mouse, WT: wild-type control (B. Strilic, unpublished data). **(C)** Simultaneous metabolic rate (MR) and abdominal body temperature (T_b_) recordings (E-Mitter Series 3000 XM-FH, 4-min resolution) of a physiological torpor event in a female C57BL/6 mouse, compared to a hypothermic individual. **(D)** MR was measured using an indirect calorimetry set-up (Heldmaier and Ruf, 1992). In **(C,D)**, shaded areas indicate the duration of “lights off.” Note in **(C)**, the steep decrease in MR and T_b_ during the middle of the dark phase, and the spontaneous arousal shortly after “lights-on,” in contrast to the slowly-decreasing MR and T_b_ in **(D)**. The hypothermic mouse in example **(D)** was removed from the calorimetry cage (arrow) and externally rewarmed without experiencing consequential damage from hypothermia. Activity counts in **(C,D)** were measured via integrated gross motor detection of the E-Mitter in the receiver field. Data are taken from Oelkrug et al. (2011).

**Figure 3:**
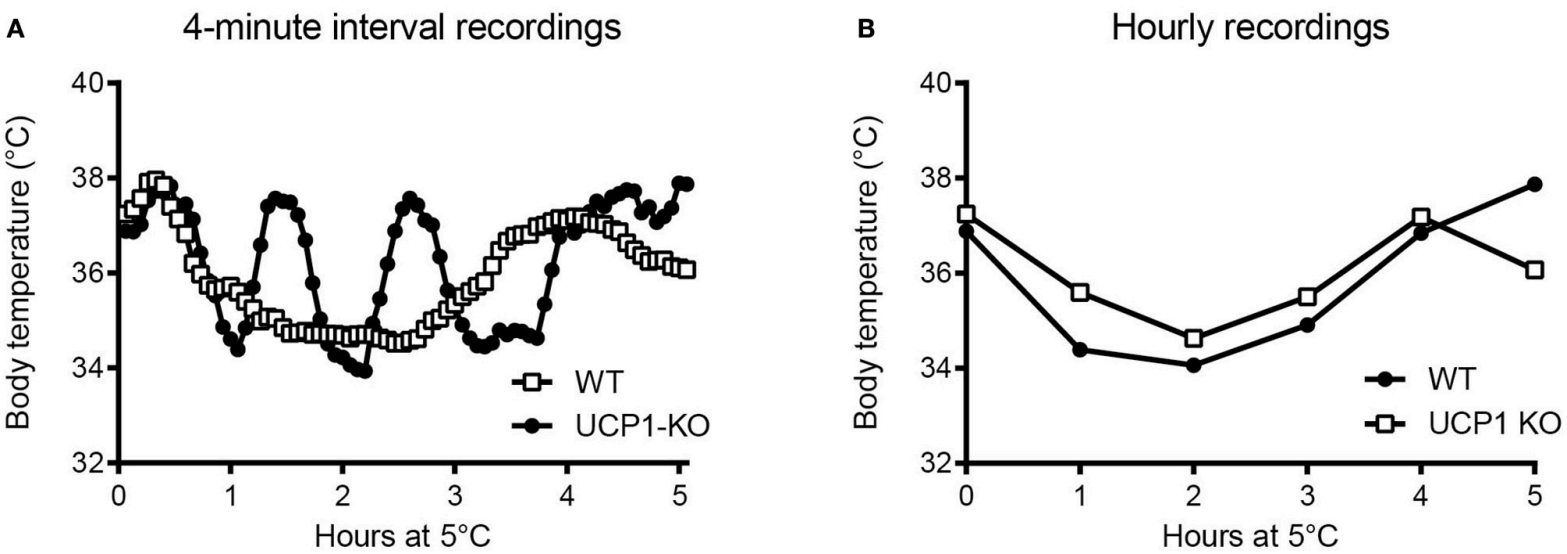
**(A)** Intra-abdominal body temperature (E-Mitter Series 3000 XM-FH) recorded every 4 min in a wild-type (WT) and an uncoupling protein-1 (UCP1)-knockout (KO) mouse previously acclimated to 18°C and acutely exposed to 5°C. Note the pronounced episodic fluctuations in body temperature of the WT mouse that are absent in the UCP1-KO mouse. Using the same data sets, we simulated hourly probing **(B)**, demonstrating resolution legacy and the information potentially missed from less-frequent sampling. Data are taken from Meyer et al. (2010).

Un-tethered, continuous monitoring of body temperature is more informative compared to single-point probing, e.g., by rectal thermometry, as periodic temperature changes are revealed, and data can be obtained in a more physiological-longitudinal framework. Some examples of valuable readouts derived from metabolic studies involving continuous body temperature measurements in unrestrained mice include circadian temperature patterns (Garami et al., 2011; Gerhart-Hines et al., 2013), ultradian-episodic (“jaggy”) events (Blessing and Ootsuka, 2016; Miyata et al., 2016), genotype- or sex-specific heterothermy (Wither et al., 2012), hyper- or hypothermic responses to pharmacological treatments (Rudaya et al., 2005; Steiner et al., 2007; Garami et al., 2011; Wanner et al., 2012; and Figure 2A), experimental fevers (Rudaya et al., 2005; Steiner et al., 2006), thermoregulatory manifestations of sepsis (Wanner et al., 2012; and Figure 2B), and body temperature patterns of the entrance into, and exit from, torpor (Oelkrug et al., 2011; Solymar et al., 2015; and Figure 2C). An example of an added value of continuous body temperature measurements in the context of cold tolerance is given in Figure 3, which depicts the data from a typical 5-h cold test performed at 4–5°C (Meyer et al., 2010). Hourly probing would have correctly identified the cold-tolerant phenotype of the uncoupling protein-1 (UCP1)-KO mouse after pre-acclimation to moderate cold, but this experimental setup would have failed to uncover the absence of periodic fluctuations in abdominal temperature, compared to wild-type. The translational value of continuous data acquisition, as compared to single probing, is also highlighted from studies in female methyl-CpG-binding protein 2 (MeCP2)-deficient mice, which initially demonstrated lower neck temperature determined by transponder. Using telemetry probes, this observation could be extended to reflect disrupted daily rhythmic patterning that mirrors impaired autonomic nervous system function and cardinal phenotypes of clinical Rett syndrome (Ward et al., 2011; Wither et al., 2012).

Some researchers also measure brain temperature in mice and other small rodents by using thermocouples, wired thermistors, or telemetry probes (Deboer et al., 1994; Romanovsky and Blatteis, 1996; Romanovsky et al., 1996; DeBow and Colbourne, 2003; Conti et al., 2006; Baracchi and Opp, 2008; Steiner et al., 2008; Ootsuka et al., 2009; Baud et al., 2013). In a telemetry probe typically used for this purpose, the XM-FH transmitter, which used to be sold by MiniMitter, the temperature sensor is located at the tip of a stainless steel cannula, which is implanted into the brain, whereas the body of the probe (which contains all the electronics) is affixed to the skull (Steiner et al., 2008).

Brain temperature is measured for different purposes and, therefore, at different locations. In the sleep field, brain temperature is often measured as a cortex temperature and used, for among other purposes, to determine the rapid-eye-movement (REM, or paradoxical) phase of sleep. In REM sleep, the activity of the cortex (and, therefore, the cortex temperature) increases. Hence, in most sleep studies, a brain probe is implanted very superficially—in the cortex, over (just above) the cortex, or even at the *dura mater* (Deboer et al., 1994; Baracchi and Opp, 2008; Baud et al., 2013). In the thermoregulation field, brain temperature is usually used as an index of deep body temperature or measured in an attempt to determine the temperature at which thermosensitive neurons, that drive autonomic thermoeffector responses, are exposed. For either purpose, the probe is implanted deeper—often into the medial anterior hypothalamus, which in most species is located close to the geometric center of the head (Romanovsky and Blatteis, 1996; Conti et al., 2006; Baracchi and Opp, 2008; Steiner et al., 2008; Baud et al., 2013). Not surprisingly, therefore, brain temperatures reported in sleep studies (cortex temperature) are often a degree or two lower than brain temperatures reported in thermoregulation studies (hypothalamic temperature).

### Infrared (IR) thermography

IR thermography, or thermal imaging, decodes the IR radiation emitted from the surface (of an animal) into a color-coded image that can be analyzed in real time or post-recording (Box 5). Of all the methods described in this paper, IR thermography is the least invasive.

Box 5 **IR thermography.**

At temperatures >0 K (−273°C), all object surfaces emit electromagnetic radiation in the infrared range of the electromagnetic spectrum (~5–15 μm). The efficacy by which energy is emitted from surfaces is called emissivity, and the emissivity coefficient (i.e., the radiation of an object in relation to that of a black body) has been determined to be 0.95–0.98 for biological materials (Cossins and Bowler, 1987). Using this information, infrared energy can be captured by infrared-sensitive cameras and processed into a thermogram, a color-coded image of the surface temperature (Speakman and Ward, 1998).

In order to retrieve reproducible data with IR thermography, important technical aspects need to be considered. For example, the intensity of emitted radiation received from an object is not just affected by its temperature, but also by the angle at which the object is viewed and the distance between the object and the camera. For assessment of living mice, most IR cameras need to be placed vertically above the mouse and within less than 1 m distance from the animal. Hence, although claiming to be non-invasive, IR measurements of live animals may require a confined space or immobilization (fixation) in order to standardize measurement conditions and the exposed surface area.

**Figure 4:**
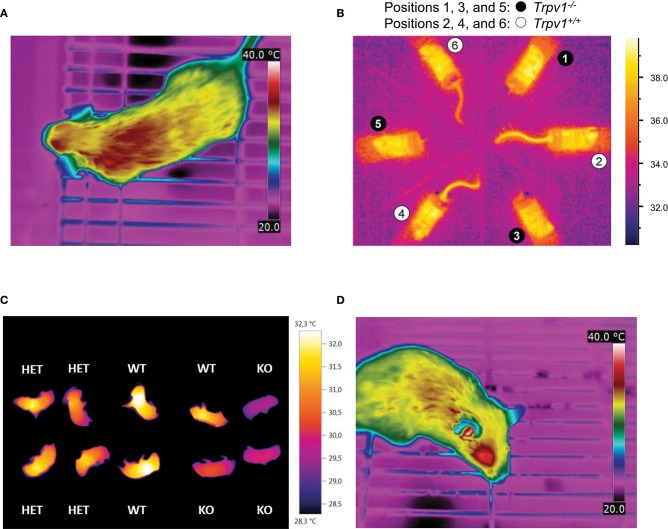
Infrared (IR) thermography in mouse metabolic studies and phenotyping. In each panel, specific color coding of radiant heat is indicated to the right. **(A)** Dorsal view from an unrestrained, conscious wild-type mouse, captured by IR thermography (T335, FLIR Systems), demonstrating heterogeneity in surface temperatures by color coding. The ambient temperature was set to 22–23°C. Image kindly provided by R. Oelkrug and J. Mittag, unpublished. **(B)** Radiant temperature from mouse tails reveals enhanced skin vasoconstriction and altered autonomous vasomotor control in transient receptor potential vanilloid-1 (Trpv1)-knockout (KO) mice compared to wild-type controls (Garami et al., 2011). The IR camera (ThermoVision A20M, FLIR Systems) was positioned above a group of confined, conscious mice inside a climatic chamber at 32°C. The mice had been previously habituated to the experimental setup by extensive handling. **(C)** Whole-body thermography in neonates (p1–p3), highlighting reduced inter-scapular skin-surface temperature in association with genetic knockout of uncoupling protein-1 (UCP-1) and impaired non-shivering thermogenesis. For the measurement, pups were placed in 6-well cell culture plates at 22–23°C ambient temperature (Maurer et al., 2015). **(D)** Lateral view from an unrestrained, conscious wild-type mouse, captured by IR thermography (T335, FLIR Systems) for specific measurement of external acoustic meatus temperature. Ambient temperature was set to 22–23°C. Image kindly provided by R. Oelkrug and J. Mittag, unpublished.

In typical applications, IR thermography does not yield deep body temperature, but measures surface (skin) temperature. As evident from individual IR thermograms (Figure 4A), surface heat radiation and, hence, surface temperature are not uniform across the body of the animal, which consists of both non-glabrous (hairy) and glabrous (non-hairy) skin (Romanovsky, 2014). Because of this heterogeneity, radiative temperature data in adult mice are often retrieved not from the entire body surface, but rather from predefined fields of view (FOVs) of a standardized size. Local surface temperature is then calculated as the average temperature across a selected FOV. Alternatively, a series of images is taken and the maximum temperature is determined from the back surface area of the animal (Gachkar et al., 2017). For rather complex technical reasons, some researchers prefer to assess the surface temperature from the frequency distribution of temperature readings across the FOV, e.g., from the warmest 10% portion (Crane et al., 2014). In general, temperature determined by IR thermography can never overestimate but only underestimate skin temperature.

One of the most robust uses of IR thermography is to assess the thermoeffector role of the glabrous skin of specialized heat-loss organs, such as the tail in mice or rats, the ear in guinea pigs and rabbits, or the hand in humans. Specifically, IR thermography is used as a measure of vasomotor tone in these organs in order to confirm the presence or absence of vasoconstriction or vasodilation (Rudaya et al., 2005; Wang et al., 2006; Garami et al., 2011; Fischer et al., 2016; Figure 4B). It should be noted, however, that in most circumstances several skin temperature-based indices (e.g., the heat loss index Romanovsky et al., 2002) are better suited for this purpose than the tail-skin temperature *per se*. As an interesting modification suitable for some specific tasks, a semi-quantitative evaluation of the vasomotor tone of the skin covering heat-loss organs can be obtained by painting it with temperature-sensitive paint (Romanovsky et al., 2002).

Another application of IR thermometry is to use the information from vasomotor tone of heat-loss organs to determine the thermoneutral zone (TNZ; see Romanovsky et al., 2002). The authoritative sources (IUPS Thermal Commission, see Bligh and Johnson, 1973; Mercer and Werner, 2001) define the TNZ as the range of ambient temperatures at which body temperature regulation is achieved only by control of sensible heat loss, i.e., without changes in metabolic heat production or evaporative heat loss. “Sensible,” or “Newtonian,” heat loss is the total heat loss due to all heat exchange mechanisms, except for evaporation. In practice, the major physiological mechanism of sensible heat loss is cutaneous vasodilation, especially in body parts that serve as heat exchangers with the environment, such as the tail of a mouse. Hence, in a subneutral (cold) environment, the tails of mice exhibit constant maximal vasoconstriction (and are difficult to see in an IR thermogram as they have nearly the same temperature as the environment). In a supraneutral (hot) environment, the tails exhibit constant maximal vasodilation (and may or may not be well-seen on thermograms, depending on the ambient temperature). In a neutral environment (i.e., within the TNZ), the tails constantly change their vasomotor tone from mild vasoconstriction to mild vasodilation, thus become intermittently from almost invisible to highly visible (see Romanovsky et al., 2002, for more detailed information; see Garami et al., 2011, and Figure 4B for examples in mice). For studying thermogenic responses by IR thermography, tight control of the ambient temperature is essential. In such studies, mice should be conscious, as most anesthetics decrease the threshold body temperature for activation of cold defenses - thus, effectively inhibiting thermogenesis (Garami et al., in press).

The utility of IR thermography in assessing the vasomotor control in mice tails is exemplified by the metabolic phenotype of mice expressing a mutant thyroid hormone receptor alpha 1 (TRa1+m). In these mice, impaired vasoconstriction of the tail arteries leads to increased heat loss in cold environments and promotes hypothermia, despite elevated brown fat activity and energy expenditure. At first sight, the hypermetabolic phenotype was “paradoxical” (Warner and Mittag, 2014), since the mutation was expected to reduce the affinity of TRa1 to thyroid hormones and, hence, was predicted to lower thermogenesis. Subsequent studies of the thermoregulatory effector organs revealed that TRa1+m mice had a greater need for adaptive thermogenesis, as their vasomotor responses were ineffective at maintaining euthermia. These studies revealed an unexpected role of thyroid hormones in thermoregulation (Sjogren et al., 2007; Warner et al., 2013). Furthermore, these findings demonstrate that hypermetabolism is not necessarily associated with high body temperature.

IR thermography is also used to assess BAT thermogenesis in rodents. For this purpose, most researchers use the difference between radiative temperatures of the inter-scapular back-skin (covers the inter-scapular BAT depots) and the lumbar back-skin (reference point) as an index of thermogenesis in the inter-scapular BAT (e.g., Marks et al., 2009; Pazos et al., 2015). We find this approach to be well-grounded. Other researchers capture the absolute values of, or the changes (from basal value) in, the inter-scapular skin temperature for the same purpose (Gerhart-Hines et al., 2013; Crane et al., 2015). This latter approach is more prone to error, but still seems to work in some cases. For example, the increase in inter-scapular skin temperature observed in wild-type mice (1.7°C) in response to the selective beta-3 adrenergic agonist CL-316,243 was absent in UCP1-KO mice, corroborating compromised BAT-function (Crane et al., 2014). In contrast, this approach did not allow for the successful quantification of BAT thermogenic capacity following beta-adrenergic stimulation or cold acclimation in a study in voles (*Microtus agrestis*; Jackson et al., 2001).

Some researchers prefer shaving the skin in trunk FOVs, whilst others do not. Under most conditions, the shaved patches give higher surface temperature readings, as any insulative effect of the pelage is eliminated. In addition, shaving lessens the confounding diffusion and reflection effects of the pelage. Of course, this is not an issue in experiments with nude pups (Figure 4C) or genetically hairless animals (Chen et al., 2013; Schulz et al., 2013; Romanovsky, 2014; Maurer et al., 2015). Whether or not shaving is required depends on the IR signal intensity and the specific goal of measurements. However, we feel that shaving should be avoided whenever possible, as it creates “thermal windows,” which may substantially increase heat loss, and thus, potentially change deep body temperature and affects the recruitment of thermoeffectors. Removing the hair also irritates the skin and can lead to inflammation, which can affect local temperatures. If repeated measurements, over a longer period of time, are required - shaving needs to be repeated frequently, as the trunk hair re-grows relatively fast (~3 weeks in adult mice Muller-Rover et al., 2001). This alters conductivity and may subsequently change the cold-stress responsiveness.

IR thermometry also has the potential of assessing deep body temperature, but not by measuring trunk-skin temperature. The recent study by Vogel et al. (2016) proposes measuring radiative temperature of the eyes in mice, as an index of their brain temperature. The external acoustic meatus is another “window to the brain” that can possibly be used for assessing brain temperature of rodents by IR thermography or thermometry (Romanovsky, 2014; Hoefig et al., 2015, 2016, and Figure 4D).

## Concluding remarks

Metabolic phenotypes of genetically modified mice can help to establish causality between a specific gene or pathway and energy metabolism *in vivo*. In this context, animal temperature is often measured to uncover thermoregulatory alterations. Here, we have summarized the main thermometry methods used for the purposes of metabolic phenotyping in mice, and have investigated their utility. It is not only the type of sensor, but also the experimental conditions, probe location and sampling frequency, that determine the biological value of the results, driving successful phenotyping.

For comprehensive phenotyping involving thermometry, the thermal environment of the animals measured must be tightly controlled. Mice are small-sized endotherms with relatively high basal metabolic costs, owing to their unfavorable surface area to volume ratio (Kleiber, 1961). Mouse pelage has relatively poor insulative capacities, making this species sensitive to cold and specifically reliant on creating and exploiting thermally advantageous microenvironments for cost-efficient body temperature regulation (Hart, 1971). Any metabolic study in mice, therefore, not only requires considerations of room temperature (gradients and variation) in the animal facility/experimental location, but also of the “operative ambient temperature” created by the animals inside the cage (Gordon et al., 1998). Factors known to affect individual metabolic costs, at otherwise constant room air temperature, are the type and amount of bedding material, nest material, and the opportunity to huddle with cagemates (Himms-Hagen and Villemure, 1992; Gordon et al., 1998; Gordon, 2004; David et al., 2013; Maher et al., 2015). Hence, control of any of these parameters potentially reduces variance in output values. Following the same reasoning, researchers should also keep in mind that any intervention or incident that disrupts the integrity of the skin/fur (e.g., shaving, surgery, alopecia) may cause specific thermoregulatory adjustments potentially contributing to extra variation in metabolic costs.

In order to provide a more complete thermoregulatory portrait of a certain genotype, it is essential that thermometry not be limited to measurements of body temperature under standard housing conditions. Rather, temperature readings should be obtained and compared at thermoneutral vs. sub-thermoneutral (cold) and supra-neutral (warm) conditions - aiming to reveal specific deficiencies in cold and heat defenses. For the same purpose, we advocate combining measurements of core body temperature with tail thermography and back thermography (Gachkar et al., 2017). In order to reveal thermoregulatory phenotypes associated with behavioral thermoregulation, temperature readings could be combined with, for example, food restriction challenges (Haemmerle et al., 2006; Meyer et al., 2010), behavioral tasks, e.g., temperature preference chambers, thermal gradients (Gordon et al., 1998; Bautista et al., 2007), or operant conditioning for thermal reward (Baldwin, 1968; Carlisle and Dubuc, 1982).

**Figure 5:**
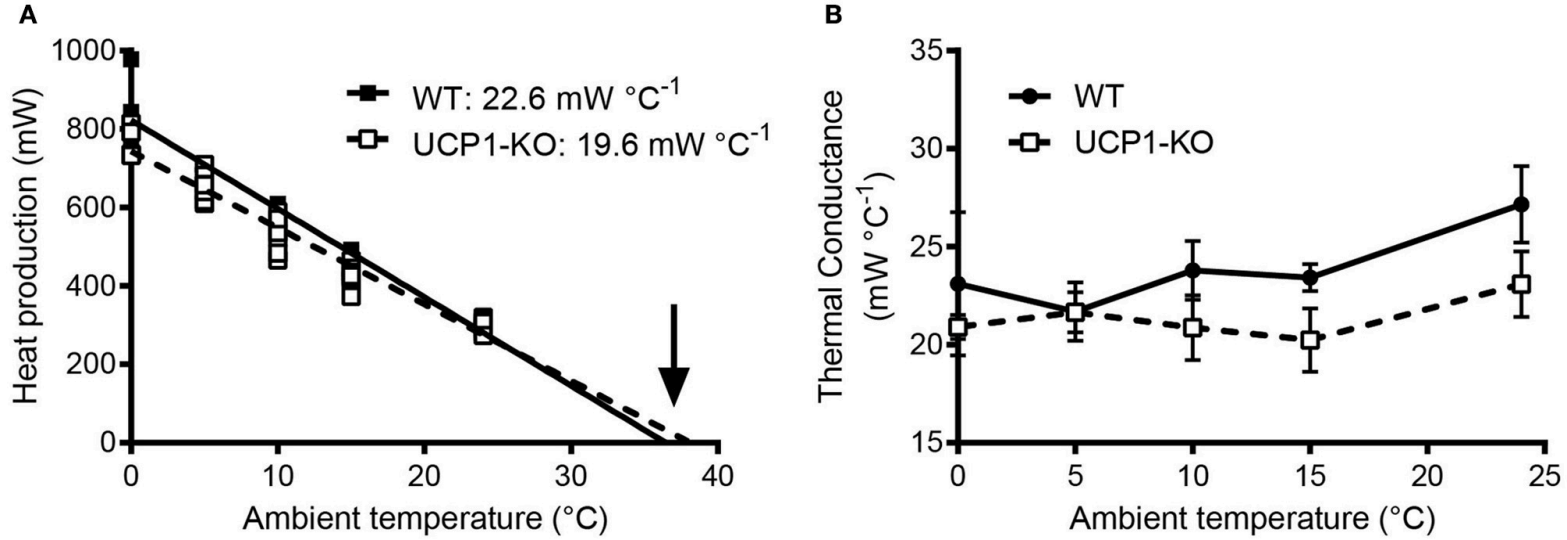
**(A)** Assessment of thermal conductance using resting heat production rates collected in wild-type and uncoupling-protein-1 (UCP1-KO)-deficient littermates at different ambient temperatures (“Scholander-Irving plot”; Scholander et al., 1950). Average slopes corresponding to predicted minimal thermal conductance (expressed as positive values) are indicated for each genotype (*p* = 0.08, *t*-test). The arrow highlights the x-axis intersection points corresponding to the predicted average body temperature during resting conditions (not different between genotypes; *p* = 0.44, *t*-test). **(B)** Calculated thermal conductance (see Box 6) involving core body temperature readings (E-Mitter Series 3000 XM-FH) in the animals shown in **(A)**. Each point indicates mean ± SD (*n* = 4–7). Both analyses, **(A,B)**, are supporting lower thermal conductance and thus an altered thermoregulatory strategy involving improved heat conservation in UCP1-KO mice. Data are taken from Meyer et al. (2010).

It is advantageous to conduct high-resolution core body temperature measurements in conjunction with measurements of metabolic rate, for the assessment for heat production rates at tightly controlled ambient temperatures. This experimental scheme not only determines the energy expenditure and associated body temperature variations, but also allows for the assessment of thermal conductance of the animals (Box 6)-a quantitative measure of the rate of heat exchange between the animal's body and the environment (McNab, 1980). In the example shown in Figure 5, thermal conductance is compared in the context of cold tolerance in UCP1-KO mice. Conductance is consistently lower in cold-acclimated UCP1-KO mice, which supports the conclusion that cold acclimation in the absence of functional BAT involves specific heat-conserving mechanisms, including improved tail vasoconstriction (Wang et al., 2006).

Box 6 **Thermal conductance.**

Metabolic phenotypes involving alterations in body temperature often depend on changes in thermogenesis (heat production), but they also reflect altered heat loss characteristics. Thermal conductance [*C*] describes the rate of heat production necessary to compensate for heat loss, i.e., the difference in temperature between the body and its surroundings (ambient temperature, T_a_). Mathematically, *C* is obtained from the slope of the linear increase in (dry) heat loss (excluding evaporation) with decreasing ambient temperature (T_a_) at temperatures cooler than thermoneutrality (Equation 1). For comparative purposes, thermal conductance should be indicated as a positive number, indicating that heat exchange is directed from the warmer to the cooler environment and not vice versa (McNab, 1980).

$$\begin{array}{l} \text{Equation}1:\text{C}={\text{HP}}^{*}{\left({\text{T}}_{\text{b}}-{\text{T}}_{\text{a}}\right)}^{-1}\\ \text{ C}=\text{thermal conductance }(\text{Watts}{}^{\circ}{\text{C}}^{-1})\\ \text{ HP}=\text{heat production }(\text{Watts},\text{W})\\ \;{\text{T}}_{\text{b}}=\text{body temperature }({}^{\circ}\text{C})\\ \;{\text{T}}_{\text{a}}=\text{ambient }(\text{air})\text{ temperature }({}^{\circ}\text{C}) \end{array}$$

Figure 5A depicts an example of this type of analysis for cold-acclimated uncoupling-protein 1 (UCP1)-knockout (KO) mice. HP was determined using indirect calorimetry. When only HP associated with resting conditions is considered, the HP-T_a_ regression extrapolates to predicted resting body temperature (“theoretical body temperature”) at zero HP, and the slope of the T_a_-HP regression elucidates minimal conductance (i.e., the minimal rate of heat production required to maintain the largest possible normothermic T_b_-T_a_ gradient; McNab, 1980). In the example shown in Figure 5A, the predicted thermal conductance (slope) is lower in UCP1-KO mice (at *p* < 0.08), and the expected core T_b_ is similar between genotypes, with a trend toward higher values in KO mice. Biologically, this indicates that UCP1-KO mice are more efficient at conserving heat.

Alternatively, HP, T_a_, and core T_b_ are measured jointly and *C* is obtained using Equation (1). Re-analysing the data and including the corresponding abdominal T_b_s measured in the same individuals confirmed that average thermal conductance in moderate cold was lower in UCP1-KO mice, (Figure 5B).

In terms of animal welfare, hyper- or hypothermic thresholds are to be established and applied carefully, as periodic deviations from “normal” temperature ranges are context-, time- and strain-dependent, and may not unequivocally indicate an acute life-threatening condition. For example, during torpor, a physiological state of metabolic depression (Jastroch et al., 2016), the abdominal body temperature of a mouse may transiently reach values that are only half a degree higher than the ambient temperature. Murine torpor commonly occurs during the early morning hours and can, in principle, be diagnosed by rectal probing. Following handling, acoustic, haptic or mechanical stimulation, a healthy mouse “alarm-arouses” and regains what is often referred to as normal metabolism and normothermy within 1–2 h, depending on the degree of metabolic depression and the ambient temperature. Of note, the classification of normothermy vs. torpor is often based on metrics of heterothermy, e.g., the amplitude of daily body temperature fluctuations (see Levesque et al., 2016, for further reading and current discussion). If a torpid mouse is left undisturbed, spontaneous arousal usually takes place by late morning to midday, but the specific onset of this process and the duration of torpor can be highly variable among strains and individuals (Dikic et al., 2008). In contrast, animals with severe energetic depletion or some metabolic deficiencies are often unable to rewarm themselves endogenously (Haemmerle et al., 2006; Oelkrug et al., 2011). In this case, 1–2 h of passive rewarming (e.g., through infrared heaters) and food and water provision are warranted (Figure 2D). Torpor frequency and associated temperature changes can be considered a metabolic phenotype and, as such, merit exploration (Jethwa et al., 2008; Swoap, 2008; Willershäuser et al., 2012).

In naive mice, a stress-induced body temperature rise can be initiated by opening the cage or even by entering the animal room. In addition, when mice are picked one-by-one from a cage, the level of distress builds up in the remaining mice kept in this cage, and the measured body temperature increases drastically from the first to the last mouse taken from the cage (Zethof et al., 1994). In this case, and in many other cases, part of the increase in body temperature can be explained by motor activity, which is directly thermogenic. Indeed, changes in the body temperature of mice follow, with some delay, changes in their motor activity (Lateef et al., 2014). In contrast, data in rats and humans suggest that rises in core temperature precede the onset of activity (Refinetti and Menaker, 1992; Decoursey et al., 1998; Ootsuka et al., 2009), as is noted to occur during ultradian cycles. While the discussion of the presence and significance of ultradian rhythms in thermogenesis is beyond the scope of this review, the technical sensitivity assessing ultradian fluctuations and temporal associations in body temperature with other physiological parameters is critically dependent on the sampling interval. In this context, we and others (Blessing and Ootsuka, 2016) recommend examination of the individual records be routinely performed before any results are averaged.

Taken together, thermometry significantly expands the metabolic phenotyping toolbox, because the interplay between heat loss and heat production can be addressed (e.g., Kaiyala et al., 2015; Abreu-Vieira et al., 2015; Fischer et al., 2016, 2017). Multi-parametric measurements involving thermometry increase the probability of revealing distinct metabolic and thermoregulatory phenotypes.

## Ethics statement

This work does not contain any animal data that was specifically collected for the purpose of publication in this manuscript. Animal data presented refer to approved experiments published elsewhere.

## Author contributions

All authors have made substantial contributions to the conception or design of the work, which includes drafting the work, and revising it critically for important intellectual content.

### Conflict of interest statement

The authors declare that the research was conducted in the absence of any commercial or financial relationships that could be construed as a potential conflict of interest.
